# 3-(4-Chloro­phenyl­diazen­yl)-1-methyl-1,4,5,6-tetra­hydro­pyridine

**DOI:** 10.1107/S1600536808018217

**Published:** 2008-06-19

**Authors:** Fiorella Meneghetti, Gabriella Bombieri, Michele Tonelli

**Affiliations:** a Institute of Pharmaceutical and Toxicological Chemistry, ‘P.Pratesi’, University of Milano, via L. Mangiagalli 25, 20133 Milano, Italy; b Department of Pharmaceutical Chemistry, University of Genova, viale Benedetto XV, 16132 Genova, Italy

## Abstract

The title compound, C_12_H_14_ClN_3_, represents the planar azoenamine tautomer. The benzene ring forms a dihedral angle of 2.5 (1)° with the azoenamine group. Electron delocalization is indicated by the values of the bond lengths in the chain. The tetra­hydro­pyridine ring adopts a half-chair conformation and the dihedral angle between the least-squares plane defined by the five coplanar C atoms and the azoenamine unit is 2.0 (1)°, while the envelope-flap C atom lies out of this plane by 0.579 (2) Å. The mol­ecular packing is governed by van der Waals inter­actions through the stacking of adjacent mol­ecules, resulting in a two-dimensional sheet structure.

## Related literature

Aryl­azoenamines are useful templates for the investigation of the role of substituents on the benzene ring in the treatment of aryl­hydrazones with acids (Canu Boido *et al.*, 1993 ▶). For related literature, see: Boido Canu *et al.* (1988 ▶); Sparatore *et al.* (1990 ▶); Cremer & Pople (1975 ▶).
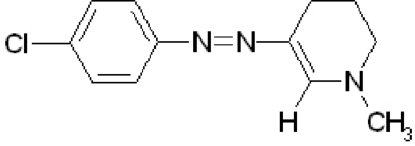


## Experimental

### 

#### Crystal data

C_12_H_14_ClN_3_
*M* *_r_* = 235.71
Triclinic, 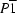

*a* = 6.251 (2) Å
*b* = 8.483 (3) Å
*c* = 11.824 (4) Å
α = 77.590 (10)°
β = 78.450 (10)°
γ = 87.01 (2)°
*V* = 599.9 (4) Å^3^
*Z* = 2
Mo *K*α radiation
μ = 0.29 mm^−1^
*T* = 293 (2) K
0.6 × 0.5 × 0.4 mm

#### Data collection

Enraf–Nonius CAD-4 diffractometer
Absorption correction: none
3028 measured reflections
2893 independent reflections
1803 reflections with *I* > 2σ(*I*)
*R*_int_ = 0.014
3 standard reflections frequency: 120 min intensity decay: <1%

#### Refinement

*R*[*F* ^2^ > 2σ(*F* ^2^)] = 0.061
*wR*(*F* ^2^) = 0.191
*S* = 1.04
2893 reflections
146 parameters
H-atom parameters constrained
Δρ_max_ = 0.33 e Å^−3^
Δρ_min_ = −0.49 e Å^−3^

### 

Data collection: *CAD-4 Software* (Enraf–Nonius, 1989 ▶); cell refinement: *CAD-4 Software*; data reduction: *XCAD4* (Harms & Wocadlo, 1995 ▶); program(s) used to solve structure: *SIR92* (Altomare *et al.*, 1994 ▶); program(s) used to refine structure: *SHELXL97* (Sheldrick, 2008 ▶); molecular graphics: *ORTEP-3 for Windows* (Farrugia, 1997 ▶); software used to prepare material for publication: *WinGX* (Farrugia, 1999 ▶).

## Supplementary Material

Crystal structure: contains datablocks I, global. DOI: 10.1107/S1600536808018217/fj2123sup1.cif

Structure factors: contains datablocks I. DOI: 10.1107/S1600536808018217/fj2123Isup2.hkl

Additional supplementary materials:  crystallographic information; 3D view; checkCIF report

## Figures and Tables

**Fig. 1.**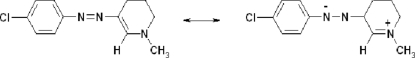: Chemical scheme of the two tautomers of 1.

**Fig. 2.**
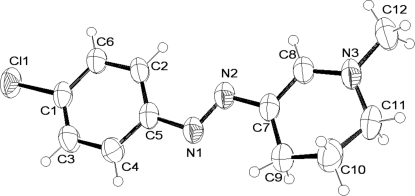
: The molecular structure of the title compound, showing atom-labelling scheme. Displacement ellipsoids are drawn at the 40% probability level.

**Fig. 3.**
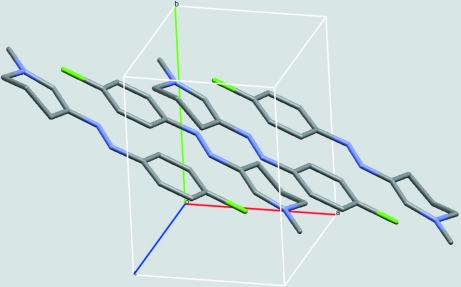
: Packing diagram of 1, showing the two-dimensional sheet structure formed by the molecular stacking.

## supplementary crystallographic information

### Comment

In order to define the influence exerted by the chloro-benzene nucleus on the yields of azoenamines, the hydrazone-hydrazoene tautomerism has been investigated by X-ray analysis of the derivative 1 (Fig. 1). The results could be an useful tool to understand the antimicrobial properties of arylazoenamine compounds (Canu Boido *et al.*, 1993). As this compound could resonate with the amphoionic structure, the X-ray analysis allowed the identification of the tautomer (Fig. 2). The extended conformation of the azoenamine skeleton is characterized by the torsion angles N3—C8—C7—N2 of 179.2 (1)°, C8—C7—N2—N1 of 179.2 (1)°, C7—N2—N1—C5 of 179.0 (1)° and N2—N1—C5—C4 of 176.9 (1)°, indicating the quite coplanarity of the aromatic ring with the azoenamine moiety. This allows a certain degree of electron delocalization, beginning at the phenyl moiety and extending through the double bond, as shown by the shortening of the bond lenghts N2—C7 of 1.361 (4)Å and C8—N3 of 1.361 (4) Å. The half chair conformation of the tetrahydropyridine ring is defined by the puckering parameter of φ2=173.3 (1)° and QT=0.426 (4)Å (Cremer & Pople, 1975). The molecular packing is governed by the van der Waals interactions through the stacking of adjacent molecules, resulting in a two-dimesional sheet structure (Fig. 3).

### Experimental

The title compound derives from the azo-coupling reaction with acids between 2-formyl-1-methylpyrrolidine and *p*-chlorophenylhydrazine (Canu Boido *et al.*, 1993). Single crystal of 1 were obtained by slow evaporation of an ethanol solution.

### Refinement

All non-H-atoms were refined anisotropically. Hydrogen atoms were introduced at calculated positions, in their described geometries and allowed to ride on the attached carbon atom with fixed isotropic thermal parameters (1.2Ueq and 1.5Ueq of the parent carbon atom for aromatic H-atoms and methyls H-atoms, respectively). The methyl H-atoms were placed with the AFIX 33 to prevent the rotational refinement.

### Crystal data

**Table:**

C_12_H_14_ClN_3_	*Z* = 2
*M**_r_* = 235.71	*F*_000_ = 248
Triclinic, *P*1	*D*_x_ = 1.305 Mg m^−^^3^
Hall symbol: -P 1	Mo *K*α radiation λ = 0.71073 Å
*a* = 6.251 (2) Å	Cell parameters from 25 reflections
*b* = 8.483 (3) Å	θ = 9–10º
*c* = 11.824 (4) Å	µ = 0.30 mm^−^^1^
α = 77.590 (10)º	*T* = 293 (2) K
β = 78.450 (10)º	Prism, red
γ = 87.01 (2)º	0.6 × 0.5 × 0.4 mm
*V* = 599.9 (4) Å^3^	

### Data collection

**Table:**

Enraf–Nonius CAD-4 diffractometer	*R*_int_ = 0.014
Radiation source: fine-focus sealed tube	θ_max_ = 28.0º
Monochromator: graphite	θ_min_ = 3.9º
*T* = 293(2) K	*h* = −8→8
Non–profiled ω/2θ scans	*k* = −11→10
Absorption correction: none	*l* = −15→0
3028 measured reflections	3 standard reflections
2893 independent reflections	every 120 min
1803 reflections with *I* > 2σ(*I*)	intensity decay: <1%

### Refinement

**Table:**

Refinement on *F*^2^	Secondary atom site location: difference Fourier map
Least-squares matrix: full	Hydrogen site location: inferred from neighbouring sites
*R*[*F*^2^ > 2σ(*F*^2^)] = 0.061	H-atom parameters constrained
*wR*(*F*^2^) = 0.191	*w* = 1/[σ^2^(*F*_o_^2^) + (0.117*P*)^2^ + 0.0064*P*] where *P* = (*F*_o_^2^ + 2*F*_c_^2^)/3
*S* = 1.04	(Δ/σ)_max_ < 0.001
2893 reflections	Δρ_max_ = 0.33 e Å^−^^3^
146 parameters	Δρ_min_ = −0.49 e Å^−^^3^
Primary atom site location: structure-invariant direct methods	Extinction correction: none

### Special details

**Table:**

Geometry. All e.s.d.'s (except the e.s.d. in the dihedral angle between two l.s. planes) are estimated using the full covariance matrix. The cell e.s.d.'s are taken into account individually in the estimation of e.s.d.'s in distances, angles and torsion angles; correlations between e.s.d.'s in cell parameters are only used when they are defined by crystal symmetry. An approximate (isotropic) treatment of cell e.s.d.'s is used for estimating e.s.d.'s involving l.s. planes.
Refinement. Refinement of *F*^2^ against ALL reflections. The weighted *R*-factor *wR* and goodness of fit *S* are based on *F*^2^, conventional *R*-factors *R* are based on *F*, with *F* set to zero for negative *F*^2^. The threshold expression of *F*^2^ > σ(*F*^2^) is used only for calculating *R*-factors(gt) *etc*. and is not relevant to the choice of reflections for refinement. *R*-factors based on *F*^2^ are statistically about twice as large as those based on *F*, and *R*- factors based on ALL data will be even larger.

### Fractional atomic coordinates and isotropic or equivalent isotropic displacement parameters (Å^2^)

**Table:**

	*x*	*y*	*z*	*U*_iso_*/*U*_eq_	
Cl1	−0.62912 (9)	0.77463 (8)	0.36467 (7)	0.0818 (3)	
N1	0.1713 (3)	0.4296 (2)	0.18348 (17)	0.0586 (5)	
N2	0.2811 (3)	0.3554 (2)	0.26078 (17)	0.0577 (5)	
N3	0.7609 (3)	0.1111 (2)	0.27394 (19)	0.0677 (6)	
C1	−0.3925 (3)	0.6722 (2)	0.3139 (2)	0.0592 (6)	
C2	−0.0779 (3)	0.5151 (3)	0.3523 (2)	0.0630 (6)	
H2	0.0076	0.4626	0.4052	0.076*	
C3	−0.3383 (4)	0.6673 (3)	0.1976 (2)	0.0670 (6)	
H3	−0.4274	0.7175	0.1459	0.080*	
C4	−0.1496 (4)	0.5873 (3)	0.1563 (2)	0.0631 (6)	
H4	−0.1117	0.5847	0.0766	0.076*	
C5	−0.0155 (3)	0.5106 (2)	0.2336 (2)	0.0562 (5)	
C6	−0.2651 (3)	0.5964 (3)	0.3933 (2)	0.0645 (6)	
H6	−0.3047	0.6002	0.4728	0.077*	
C7	0.4609 (3)	0.2732 (2)	0.2171 (2)	0.0585 (6)	
C8	0.5789 (3)	0.1961 (3)	0.2987 (2)	0.0600 (6)	
H8	0.5286	0.2036	0.3769	0.072*	
C9	0.5288 (4)	0.2646 (3)	0.0908 (2)	0.0698 (6)	
H9A	0.6038	0.3629	0.0471	0.084*	
H9B	0.4007	0.2552	0.0581	0.084*	
C10	0.6793 (5)	0.1200 (4)	0.0783 (3)	0.0841 (8)	
H10A	0.5929	0.0225	0.1014	0.101*	
H10B	0.7492	0.1302	−0.0039	0.101*	
C11	0.8530 (4)	0.1044 (3)	0.1526 (3)	0.0780 (8)	
H11A	0.9306	0.0027	0.1504	0.094*	
H11B	0.9574	0.1908	0.1194	0.094*	
C12	0.8856 (4)	0.0404 (3)	0.3635 (3)	0.0819 (8)	
H12A	0.8063	0.0531	0.4394	0.123*	
H12B	1.0237	0.0936	0.3467	0.123*	
H12C	0.9096	−0.0724	0.3637	0.123*	

### Atomic displacement parameters (Å^2^)

**Table:**

	*U*^11^	*U*^22^	*U*^33^	*U*^12^	*U*^13^	*U*^23^
Cl1	0.0537 (4)	0.0776 (5)	0.1070 (6)	0.0248 (3)	−0.0085 (3)	−0.0163 (4)
N1	0.0465 (9)	0.0571 (10)	0.0711 (12)	0.0101 (7)	−0.0116 (8)	−0.0129 (9)
N2	0.0441 (9)	0.0510 (9)	0.0756 (12)	0.0059 (7)	−0.0095 (8)	−0.0111 (8)
N3	0.0461 (9)	0.0618 (11)	0.0926 (15)	0.0147 (8)	−0.0137 (10)	−0.0136 (10)
C1	0.0427 (10)	0.0480 (11)	0.0835 (17)	0.0082 (8)	−0.0106 (10)	−0.0101 (10)
C2	0.0450 (11)	0.0723 (14)	0.0733 (16)	0.0150 (10)	−0.0188 (10)	−0.0154 (11)
C3	0.0579 (12)	0.0606 (13)	0.0815 (17)	0.0182 (10)	−0.0213 (12)	−0.0102 (12)
C4	0.0609 (13)	0.0565 (12)	0.0695 (14)	0.0132 (10)	−0.0151 (11)	−0.0089 (10)
C5	0.0435 (10)	0.0486 (11)	0.0755 (15)	0.0036 (8)	−0.0115 (10)	−0.0116 (10)
C6	0.0494 (11)	0.0736 (14)	0.0701 (14)	0.0142 (10)	−0.0117 (10)	−0.0176 (11)
C7	0.0439 (10)	0.0527 (11)	0.0768 (15)	0.0064 (9)	−0.0087 (10)	−0.0131 (10)
C8	0.0459 (10)	0.0569 (12)	0.0748 (14)	0.0070 (9)	−0.0118 (10)	−0.0101 (10)
C9	0.0559 (12)	0.0736 (15)	0.0780 (16)	0.0154 (11)	−0.0117 (11)	−0.0170 (12)
C10	0.0721 (16)	0.0881 (19)	0.096 (2)	0.0231 (14)	−0.0126 (14)	−0.0356 (16)
C11	0.0522 (13)	0.0751 (16)	0.106 (2)	0.0177 (11)	−0.0069 (13)	−0.0291 (15)
C12	0.0600 (14)	0.0723 (16)	0.111 (2)	0.0158 (12)	−0.0264 (14)	−0.0085 (14)

### Geometric parameters (Å, °)

**Table:**

Cl1—C1	1.743 (2)	C6—H6	0.9300
N1—N2	1.289 (3)	C7—C8	1.366 (3)
N1—C5	1.414 (3)	C7—C9	1.484 (4)
N2—C7	1.363 (3)	C8—H8	0.9300
N3—C8	1.331 (3)	C9—C10	1.521 (3)
N3—C11	1.447 (3)	C9—H9A	0.9700
N3—C12	1.448 (3)	C9—H9B	0.9700
C1—C3	1.358 (4)	C10—C11	1.512 (4)
C1—C6	1.383 (3)	C10—H10A	0.9700
C2—C6	1.383 (3)	C10—H10B	0.9700
C2—C5	1.388 (4)	C11—H11A	0.9700
C2—H2	0.9300	C11—H11B	0.9700
C3—C4	1.385 (3)	C12—H12A	0.9600
C3—H3	0.9300	C12—H12B	0.9600
C4—C5	1.398 (3)	C12—H12C	0.9600
C4—H4	0.9300		
			
N2—N1—C5	112.81 (19)	N3—C8—H8	117.8
N1—N2—C7	115.1 (2)	C7—C8—H8	117.8
C8—N3—C11	119.8 (2)	C7—C9—C10	110.2 (2)
C8—N3—C12	121.7 (2)	C7—C9—H9A	109.6
C11—N3—C12	118.06 (19)	C10—C9—H9A	109.6
C3—C1—C6	121.5 (2)	C7—C9—H9B	109.6
C3—C1—Cl1	119.29 (18)	C10—C9—H9B	109.6
C6—C1—Cl1	119.2 (2)	H9A—C9—H9B	108.1
C6—C2—C5	121.1 (2)	C11—C10—C9	112.6 (2)
C6—C2—H2	119.5	C11—C10—H10A	109.1
C5—C2—H2	119.5	C9—C10—H10A	109.1
C1—C3—C4	119.7 (2)	C11—C10—H10B	109.1
C1—C3—H3	120.2	C9—C10—H10B	109.1
C4—C3—H3	120.2	H10A—C10—H10B	107.8
C3—C4—C5	120.5 (2)	N3—C11—C10	111.89 (19)
C3—C4—H4	119.7	N3—C11—H11A	109.2
C5—C4—H4	119.7	C10—C11—H11A	109.2
C2—C5—C4	118.33 (19)	N3—C11—H11B	109.2
C2—C5—N1	125.3 (2)	C10—C11—H11B	109.2
C4—C5—N1	116.3 (2)	H11A—C11—H11B	107.9
C2—C6—C1	118.9 (2)	N3—C12—H12A	109.5
C2—C6—H6	120.6	N3—C12—H12B	109.5
C1—C6—H6	120.6	H12A—C12—H12B	109.5
N2—C7—C8	115.1 (2)	N3—C12—H12C	109.5
N2—C7—C9	124.0 (2)	H12A—C12—H12C	109.5
C8—C7—C9	120.91 (19)	H12B—C12—H12C	109.5
N3—C8—C7	124.5 (2)		

## References

[bb1] Altomare, A., Cascarano, G., Giacovazzo, C., Guagliardi, A., Burla, M. C., Polidori, G. & Camalli, M. (1994). *J. Appl. Cryst.* **27**, 435.

[bb2] Boido Canu, C., Boido, V., Sparatore, F., Sparatore, A., Susanna, V., Russo, S., Canicola, M. L. & Marmo, E. (1988). *Farmaco Ed. Sci.* **43**, 819–837. 3266484

[bb3] Canu Boido, C., Boido, V., Sparatore, F., Sparatore, A., Bombieri, G., Benetollo, F., Debbia, E. & Pesce Schito, A. (1993). *Il Farmaco*, **48**, 749–775. 8373502

[bb4] Cremer, D. & Pople, J. A. (1975). *J. Am. Chem. Soc.* **97**, 1354–1358.

[bb5] Enraf–Nonius (1989). *CAD-4 Software* Enraf–Nonius, Delft, The Netherlands.

[bb6] Farrugia, L. J. (1997). *J. Appl. Cryst.* **30**, 565.

[bb7] Farrugia, L. J. (1999). *J. Appl. Cryst.* **32**, 837–838.

[bb8] Harms, K. & Wocadlo, S. (1995). *XCAD4* University of Marburg, Germany.

[bb9] Sheldrick, G. M. (2008). *Acta Cryst.* A**64**, 112–122. 10.1107/S0108767307043930 18156677

[bb10] Sparatore, A., Canu Boido, C., Boido, V., Sparatore, F., Debbia, E. & Pesce Schito, A. (1990). *Il Farmaco*, **45**, 867–877. 8373502

